# Evaluation of the Indications for Sentinel Node Biopsy in Early-Stage Melanoma with the Advent of Adjuvant Systemic Therapy: An International, Multicenter Study

**DOI:** 10.1245/s10434-022-11761-4

**Published:** 2022-05-13

**Authors:** Marc D. Moncrieff, Serigne N. Lo, Richard A. Scolyer, Martin J. Heaton, Jenny P. Nobes, Andrew P. Snelling, Michael J. Carr, Carolyn Nessim, Ryckie Wade, A. Howard Peach, Rumi Kisyova, Jennifer Mason, Ewan D. Wilson, Grant Nolan, Rowan Pritchard Jones, Vernon K. Sondak, John F. Thompson, Jonathan S. Zager

**Affiliations:** 1 Department of Plastic and Reconstructive Surgery, Norfolk and Norwich University Hospital NHS Trust, Norwich, UK; 2 Norwich Medical School, University of East Anglia, Norwich, UK; 3 Melanoma Institute of Australia, University of Sydney, Sydney, Australia; 4 Royal Prince Alfred Hospital, Sydney, NSW Australia; 5 NSW Health Pathology, Sydney, NSW Australia; 6 Faculty of Medicine and Health, The University of Sydney, Sydney, NSW Australia; 7 H. Lee Moffitt Cancer Center, Tampa, Florida USA; 8 Ottawa Hospital, Ottawa, Canada; 9 Leeds Teaching Hospitals NHS Trust, Leeds, UK; 10 North Bristol Hospital NHS Trust, Bristol, UK; 11 St. Helens and Knowsley NHS Trust, Liverpool, UK

## Abstract

**Background:**

Patients presenting with early-stage melanoma (AJCC pT1b-pT2a) reportedly have a relatively low risk of a positive SNB (~5–10%). Those patients are usually found to have low-volume metastatic disease after SNB, typically reclassified to AJCC stage IIIA, with an excellent prognosis of ~90% 5-year survival. Currently, adjuvant systemic therapy is not routinely recommended for most patients with AJCC stage IIIA melanoma. The purpose was to assess the SN-positivity rate in early-stage melanoma and to identify primary tumor characteristics associated with high-risk nodal disease eligible for adjuvant systemic therapy

**Methods:**

An international, multicenter retrospective cohort study from 7 large-volume cancer centers identified 3,610 patients with early primary cutaneous melanomas 0.8–2.0 mm in Breslow thickness (pT1b-pT2a; AJCC 8th edition). Patient demographics, primary tumor characteristics, and SNB status/details were analyzed.

**Results:**

The overall SNB-positivity rate was 11.4% (412/3610). Virtually all SNB-positive patients (409/412; 99.3%) were reclassified to AJCC stage IIIA. Multivariate analysis identified age, T-stage, mitotic rate, primary site and subtype, and lymphovascular invasion as independent predictors of sentinel node status. A mitotic rate of >1/mm^2^ was associated with a significantly increased SN-positivity rate and was the only significant independent predictor of high-risk SNB metastases (>1 mm maximum diameter).

**Conclusions:**

The new treatment paradigm brings into question the role of SNB for patients with early-stage melanoma. The results of this large international cohort study suggest that a reevaluation of the indications for SNB for some patients with early-stage melanoma is required.

Early-stage invasive melanoma can be defined as primary tumors with Breslow thickness 0.8-2.0 mm or T-stage pT1b-pT2a according to the TNM classification system (AJCC 8th edition Staging System).^1^ The current standard of care for early-invasive melanoma is to stage the patients with a sentinel node biopsy (SNB).^2^ The incidence of sentinel node metastasis from early-stage invasive primary cutaneous melanoma is low, previously reported at 5-10%.^3^ In contrast, the risk of nodal metastasis from advanced primary cutaneous melanoma (Breslow thickness >4 mm, pT4b) is 35-50%. The outcomes of the MSLT-1,^4^ MSLT-2,^5^ and DeCOG^6^ studies, in addition to the maturation of data from recent adjuvant systemic therapy trials,^7,8^ have embedded the role of SNB for accurately staging patients with cutaneous melanoma, whilst simultaneously shifting the treatment paradigm to identifying those eligible for adjuvant systemic therapy, rather than completion lymph node dissection. Nearly all patients with early-stage melanoma who are subsequently found to be SNB-positive (SN+) still have an excellent prognosis, with 5-year survival approaching 90%.^1^ These patients are mapped to the AJCC Stage IIIA category.^1^ Adjuvant systemic therapy is usually not routinely recommended for this group, particularly if the maximum diameter of the tumor deposit is 1 mm or less.^2^ Accordingly, the low incidence of SN-positivity and the very limited role of adjuvant systemic therapy even when SN-positivity is diagnosed brings into question the role of SNB for patients with early-stage melanoma.

The dual purposes of this study were to identify patients with early-stage primary melanomas (AJCC pT1b-pT2a) who were more likely to have micrometastatic disease in a SN and to identify primary tumor characteristics predictive of patients with higher-risk nodal disease, who might potentially benefit from adjuvant systemic therapy.

## Methods

Central regulatory approval for this study was granted by the UK NHS Health Research Authority (IRAS ID: 284808). A consortium of seven, high-volume cancer centers from Australia, UK, and North America with prospective, institutionally maintained melanoma databases agreed to collaborate in this study. All centers had similar referral guidelines for SNB based on the current AJCC staging criteria and routinely offered SNB to patients with primary melanomas stage pT1b and above.^1,9^ All centers routinely undertake internal central pathology review by dedicated dermatopathologists prior to offering SNB. The primary inclusion criteria for this study were adult patients, aged 18 years or older, with pathological stage pT1b and pT2a primary cutaneous melanomas who underwent SNB between 2005 and 2020.

Standard patient demographic data and tumor characteristics were recorded. Mitotic rate (MR), defined as mitoses per square millimeter, was measured as per the AJCC criteria using the “hot-spot” method.^1^ Details of the sentinel node biopsy report included nodal status and, for the SN+ cases, N-stage, maximum size of tumor deposit.^10^ Historic completion lymph node dissection (CLND) data were not used in the analysis due to the lack of consistency for the indications for the procedure across the centers during the study period and the irreconcilable bias of several centers participating in the MSLT-2 study at the same time.^5^

### Statistical Analysis

Pseudoanonymised data were analyzed using Jamovi software (Version 1.6, Sydney, Australia https://www.jamovi.org) and R-Studio (version 1.3.1093, Boston, MA), both running R-language (version 3.6, https://cran.r-project.org/). Patients’ characteristics and histopathological parameters were summarized using descriptive statistics stratified by SN status. Differences between groups were tested using Kruskal-Wallis or Student’s *t*-tests as appropriate for continuous variables and chi-squared tests for categorical variables. Subgroup analyses were performed to assess the incidence of SN+ within pT stage and MR categories.

A model to predict the likelihood being of SN+ for individual patients was built using multivariable logistic regression and a set of factors clinically selected. Factors predicting high risk of SN tumor burden based on the maximum tumor deposit size among patients with positive SNBs were analyzed using multivariable logistic regression.

## Results

Seven centers identified 3610 patients meeting the inclusion criteria. The data was collated from a period ranging from 2005–2020, inclusive.

### Sentinel Node Status and Primary Tumor Characteristics

Table 1 provides a full comparison of patients’ and primary tumor characteristics, stratified by the primary outcome variable, SN status. The overall incidence of SN+ was 11.4% (412/3610; 95% confidence interval [CI]: 10.4-12.5%) with a range of 8.3% to 18.3% (X^2^ = 14.14, *p* = 0.028) between the treatment centers. SN status was significantly associated with the following variables: age, primary site location, melanoma subtype, pT stage, mitotic rate, and the presence of lymphovascular invasion. Lymphovascular invasion was associated with a 28.6% risk of SN+, but the incidence of this characteristic was 2.0% (70/3,481 cases). The incidence of SN+ was significantly correlated with increasing mitotic rate and increasing Breslow thickness but a significant inverse correlation was seen with age. The median Breslow thickness in the SN+ was 1.3 mm compared with 1.2 mm in the SN− group and the means were 1.38 mm and 1.28 mm, respectively (t-test, df = 2608, *p* < 0.001; the clinical effect size (Cohen’s d) = 0.33 (95% CIs: 0.23-0.43). For the pT1b (Breslow ≤1 mm) subgroup, the SN+ rate was 7.6% and the pT2a (Breslow 1.01-2 mm) subgroup was 12.8%.

**Table 1:** Dataset summary, stratified by sentinel node status

Dependent: sentinel node status						
Dependent (N)^a^		Negative N = 3,198 (%)	Positive N = 410 (%)	Incidence Positive (%)	Total (%)	Test statistic
Center	Sydney	1400 (43.8)	180 (43.7)	11.4%	1580 (43.8)	X^2^(6) = 14.14, *p* = 0.028^b^
(N = 3610)	Bristol	227 (7.1)	36 (8.7)	13.7%	263 (7.3)	
	Leeds	196 (6.1)	28 (6.8)	12.5%	224 (6.2)	
	Liverpool	134 (4.2)	30 (7.3)	18.3%	164 (4.5)	
	Moffitt	483 (15.1)	45 (10.9)	8.5%	528 (14.6)	
	Norwich	550 (17.2)	70 (17.0)	11.3%	620 (17.2)	
	Ottawa	208 (6.5)	23 (5.6)	10.0%	231 (6.4)	
Age (N = 3610)	Median (IQR)	59.0 (48.0-68.0)	54.0 (44.0-65.0)	–	58.0 (47.0-68.0)	F1,3608 = 20.11, *p* < 0.001^c^
Gender	F	1490 (46.6)	177 (43.0)	10.6%	1667 (46.2)	X^2^(1) = 1.94, *p* = 0.16^b^
(N = 3610)	M	1708 (53.4)	235 (57.0)	12.1%	1943 (53.8)	
Primary site	Torso	1172 (36.6)	172 (41.7)	12.8%	1344 (37.2)	X^2^(3) = 22.82, *p* < 0.001^b^
(N = 3610)	Head/Neck	448 (14.0)	57 (13.8)	11.3%	505 (14.0)	
	Upper Limb	793 (24.8)	60 (14.6)	7.0%	853 (23.6)	
	Lower Limb	785 (24.5)	123 (29.9)	13.5%	908 (25.2)	
Melanoma subtype	SSM	2291 (71.6)	311 (75.5)	12.0%	2602 (72.1)	X^2^(4) = 12.94, *p* = 0.012^b^
(N = 3610)	Nodular	426 (13.3)	56 (13.6)	11.6%	482 (13.4)	
	Acral	38 (1.2)	9 (2.2)	19.1%	47 (1.3)	
	Lentigo Maligna	122 (3.8)	5 (1.2)	3.9%	127 (3.5)	
	Other	321 (10.0)	31 (7.5)	8.8%	352 (9.8)	
Breslow (N = 3610)	Median (IQR)	1.2 (1.0-1.5)	1.3 (1.1-1.7)	–	1.2 (1.0-1.5)	F1,3608 = 39.54, *p* < 0.001^c^
T stage	T1b	912 (28.5)	75 (18.2)	7.6%	987 (27.3)	X^2^(1) = 19.54, *p* < 0.001^b^
(N = 3610)	T2a	2286 (71.5)	337 (81.8)	12.8%	2623 (72.7)	
MR (N = 3610)	Median (IQR)	2.0 (1.0-3.0)	2.0 (1.0-4.0)	–	2.0 (1.0-3.0)	F1,3608 = 32.98, *p* < 0.001^c^
*MR category*						
(N = 3610)	0 per mm^2^	556 (17.4)	30 (7.3)	5.2%	586 (16.2)	X^2^(2) = 34.43, *p* < 0.001^b^
	1 per mm^2^	899 (28.1)	105 (25.5)	10.5%	1004 (27.8)	
	>1 per mm^2^	1743 (54.5)	277 (67.2)	13.7%	2020 (56.0)	
MR risk*	Low	1455 (45.5)	135 (32.8)	8.5%	1590 (44.0)	X^2^(1) = 24.00, *p* < 0.001^b^
(N = 3610)	High	1743 (54.5)	277 (67.2)	13.7%	2020 (56.0)	
LVI	Absent	3027 (98.4)	384 (95.0)	11.3%	3411 (98.0)	X^2^(1) = 20.04, *p* < 0.001^b^
(N = 3481)	Present	50 (1.6)	20 (5.0)	28.6%	70 (2.0)	
Perineural invasion	Absent	1422 (98.8)	195 (100.0)	12.1%	1617 (98.9)	X^2^(1) = 2.46, *p* = 0.12^b^
(N = 1635)	Present	18 (1.2)	0 (0.0)	0.0%	18 (1.1)	
Regression (N = 2942)	Absent	1401 (53.8)	189 (56.1)	11.9%	1590 (54.0)	X^2^(1) = 0.64, *p* = 0.42^b^
	Present	1204 (46.2)	148 (43.9)	10.9%	1352 (46.0)	
Ulceration*	Absent	851 (93.3)	70 (93.3)	8.2%	3513 (97.3)	X^2^(1) = 0.00, *p* = 0.99^b^
(N = 987)	Present	61 (6.7)	5 (6.7)	8.2%	97 (2.7)	
N stage	N0	3198 (100.0)	–	–	3198 (88.6)	n/a
(N = 3610)	N1a	–	328 (79.6)	–	328 (9.1)	
	N2a	–	81 (19.7)	–	81 (2.2)	
	N3a	–	3 (0.7)	–	3 (0.1)	

^a^ Number of non-missing values

^b^ Pearson (degrees of freedom)

^c^ Kruskal-Wallis

*IQR* interquartile range; *LVI* lymphovascular invasion; *MR* mitotic rate (per mm^2^)

MR Risk: High = >1 mitoses per mm^2^; Low = 0-1 mitoses per mm^2^

^*^Ulceration status: pT1 tumors only

Correlation matrix assessment of Breslow thickness and MR demonstrated a significant, positive association between the two variables (Pearson’s r = 0.233, *p* < 0.001). The subgroup analyses for predictors of SN status are shown in Table 2, focusing on pT stage and MR categories. The rate of SN+ rate ranged from 4.5% to 9.8% across the MR categories in the pT1b group (χ^2^(2) = 6.19, *p* = 0.045) and from 5.5% to 14.7% in the pT2a group (χ^2^(2) = 21.6, *p* < 0.001). Table 2 shows that 67.2% of all SN+ arose from primary melanomas with a MR >1/mm^2^, compared with 7.3% of melanomas with MR of 0/mm^2^ (χ^2^ = 34.4, df = 2, *p* < 0.001). Similarly, 81.8% of SN+ were from primary melanomas in the T2 group, compared with 18.2% in the T1 group (χ^2^ = 19.54, df = 1, *p* < 0.001; Table 1). Likelihood ratios were calculated for subgroups of patients stratified by T-stage and MR category or MR category alone (Table 2). A MR of 0/mm^2^ decreased the likelihood of SN+ by a factor of 0.37 (95% CI: 0.20-0.67) in the pT1b group and 0.46 (95% CI: 0.29-0.72) in the pT2a subgroup, and by a factor of 0.42 (0.29-0.60) overall.

**Table 2:** Rates sentinel node metastasis by stage and mitotic rate category

Stage	Mitotic rate category	N (% stage)	SN+,N (rate, %)*	Likelihood ratio (95% CIs)	High risk SN+, N (rate, %)**	Likelihood ratio (95% CIs)	Statistic^a,b^
T1b	*0/mm*^*2*^	243 (24.6%)	11 (4.5%)	0.37 (0.20-0.67)	1 (0.4%)	0.11 (0.02-0.78)	
	*1/mm*^*2*^	336 (34.0%)	24 (7.1%)	0.60 (0.40-0.89)	6 (1.8%)	0.49 (0.22-1.07)	χ^2^ = 1.70;* p* = 0.19^a^
	*>1/mm*^*2*^	408 (41.3%)	40 (9.8%)	0.84 (0.62-1.15)	11 (2.7%)	0.74 (0.42-1.32)	χ^2^ = 5.87;* p* = 0.015^a^
	*Total*	987 (27.3%)	75 (7.6%)		18 (1.8%)		χ^2^(2) = 6.19; *p* = 0.045^b^
T2a	*0/mm*^*2*^	343 (13.1%)	19 (5.5%)	0.46 (0.29-0.72)	5 (1.5%)	0.40 (0.17-0.94)	
	*1/mm*^*2*^	668 (25.5%)	81 (12.1%)	1.07 (0.87-1.32)	19 (2.8%)	0.78 (0.51-1.19)	χ^2^ = 11.0;* p* = 0.001^a^
	*>1/mm*^*2*^	1612 (61.5%)	237 (14.7%)	1.34 (1.22-1.47)	88 (5.5%)	1.55 (1.37-1.75)	χ^2^ = 20.9; *p* < 0.001^a^
	*Total*	2623 (72.7%)	337 (12.8%)		112 (6.9%)		χ^2^(2) = 21.6, *p* < 0.001^b^
T1b-T2a	*0/mm*^*2*^	586 (16.2%)	30 (5.1%)	0.42 (0.29-0.60)	6 (1.0%)	0.23 (0.10-0.50)	
	*1/mm*^*2*^	1004 (27.8%)	105 (10.5%)	0.91 (0.76-1.08)	25 (2.5%)	0.56 (0.39-0.80)	χ^2^ = 13.6; *p* < 0.001^a^
	*>1/mm*^*2*^	2020 (56.0%)	277 (13.7%)	1.23 (1.15-1.33)	99 (4.9%)	1.68 (1.52-1.87)	χ^2^ = 32.2; *p* < 0.001^a^
	*Total*	3610 (100.0%)	412 (11.4%)		130 (3.6%)		χ^2^(2) = 34.4, *p* < 0.001^b^

^a^Pearson chi-square test with one degrees of freedom

^b^Chi-square test for trend with two degrees of freedom

^*^SN+ = positive sentinel node; **positive sentinel node with maximum tumor deposit size >1 mm

**Fig. 1:**
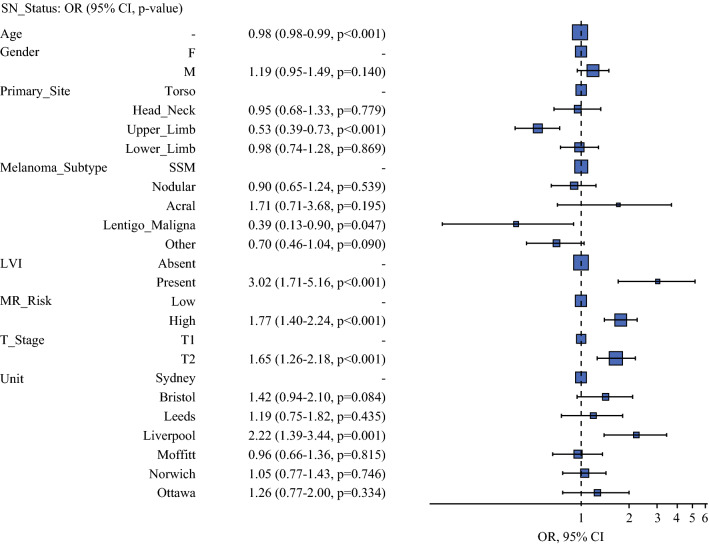
Binomial multivariable analysis of predictors for sentinel node status as an odds ratio plot. Increased odds of sentinel node metastasis to the right of the dotted line of null effect

Multivariable logistic regression analysis identified age, primary site, Breslow thickness, MR, and lymphovascular invasion as significant independent predictors of sentinel node status. Figure 1 represents the results of the binomial multivariable analysis as an odds ratio plot. T2 stage, increasing mitotic rate and lymphovascular invasion were significantly associated with increased odds of SN+.

### Nodal Risk

The incidences of N1, N2, and N3 disease were 79.6%, 19.7%, and 0.7%, respectively, meaning that 99.3% of the SN+ patients were reclassified to AJCC stage IIIA following their SNB. The current NCCN guidelines^2^ suggest that SN+ classified with AJCC stage IIIA disease and a maximum deposit size greater than 1.0 mm may be considered for adjuvant systemic therapy. Accordingly, this definition was taken as the cut-point for classifying SN+ disease as low-risk (≤1 mm) or high-risk (>1 mm). Table 2 shows that the overall incidence of high-risk SN+ disease was 3.6% (130/3610). The high-risk SN+ rate in the pT1b group was 1.8% (18/987) and 6.9% (112/2623) in the pT2a group. Binomial logistic regression analysis was performed for 404 complete record sets, which identified MR >1/mm^2^ as the only primary tumor characteristic that was a significant independent predictor of high-risk SN+ disease (odds ratio [OR] = 1.95; 95% CI: 1.20-3.23, *p* = 0.008). Of the cohort with SN+ melanomas, 35.8% (98/274) of patients had primaries with MR >1/mm^2^ and high-risk SN+ disease compared with 21.5% (28/130) of patients with MR 0-1/mm^2^ and high-risk SN+ disease (X^2^(1) = 8.32, *p* = 0.004) (Table 3).

**Table 3:** Rates of high-risk sentinel node metastasis (where tumor deposit size >1 mm in maximum diameter) by patient and primary tumor characteristics

		Low N = 278 (%)	High N = 126 (%)	Total N = 404 (%)	Test statistic	Odds ratio multivariable* (95% CIs, statistic)
Age	Median (IQR)	56.0 (44.2-66.0)	52.0 (43.2-61.8)	54.0 (44.0-65.0)	F1,402 = 2.81, *p* = 0.09^c^	0.99 (0.97-1.00, *p* = 0.062)
Gender	F	124 (44.6)	49 (38.9)	173 (42.8)	X^2^(1) = 1.16, *p* = 0.28^b^	*Referent*
	M	154 (55.4)	77 (61.1)	231 (57.2)		1.31 (0.84-2.04, *p* = 0.234)
Breslow	Median (IQR)	1.3 (1.1-1.7)	1.4 (1.2-1.8)	1.3 (1.1-1.7)	F1,402 = 1.22, *p* = 0.27^c^	–
T Stage	T1	57 (20.5)	18 (14.3)	75 (18.6)	χ^2^(1) = 2.22, *p* = 0.14^b^	*Referent*
	T2	221 (79.5)	108 (85.7)	329 (81.4)		1.38 (0.77-2.55, *p* = 0.292)
MR	Median (IQR)	2.0 (1.0-4.0)	3.0 (2.0-5.0)	2.0 (1.0-4.0)	F1,402 = 11.87, *p* < 0.001^c^	–
MR Risk	Low	102 (36.7)	28 (22.2)	130 (32.2)	χ^2^(1) = 8.32, *p* = 0.004^b^	*Referent*
	High	176 (63.3)	98 (77.8)	274 (67.8)		1.95 (1.20-3.23, *p* = 0.008)
LVI	Present	11 (4.0)	9 (7.1)	20 (5.0)	χ^2^(1) = 1.87, *p* = 0.17^b^	*Referent*
	Absent	267 (96.0)	117 (92.9)	384 (95.0)		1.90 (0.73-4.82, *p* = 0.177)

^a^ Number of non-missing values

^b^ Pearson (degrees of freedom)

^c^ Kruskal-Wallis

IQR interquartile range; LVI lymphovascular invasion; MR mitotic rate (per mm^2^)

MR Risk: High = >1 mitoses per mm^2^; Low = 0-1 mitoses per mm^2^

^*^Binomial multivariable analysis stratified by age, sex, primary site, T-stage, MR risk, and LVI

## Discussion

Since the publication of the MSLT-1 data, the therapeutic utility of adjuvant systemic therapy for stage III melanoma has been increasingly accepted internationally.^2^ Furthermore, the MSLT-2 and DeCOG studies have shown that CLND does not confer a significant survival advantage for SN+ patients and it is no longer routinely recommended.^5,6^ Before this, the main focus of the debate regarding sentinel node biopsy was the threshold (i.e., primary pT-stage, Breslow thickness, ulceration status, mitotic rate) at which the procedure should be offered, given an arbitrary predicted positive test rate of 5%.^1,9,11^ Some academic centers published algorithms to provide a further, nuanced selection criterion,^12–16^ and others debated whether SNB should be offered at all.^17,18^ Ultimately, the role of SNB for accurately staging patients with cutaneous melanoma remains, but the primary rationale for offering the procedure has altered to identifying those most likely to benefit from adjuvant systemic therapy. The focus of the current study is to reappraise those who are *least* likely to benefit from being staged by the procedure, particularly if the outcome does not alter subsequent management of the patient. The focus of the current analysis has been to assess the early-stage invasive melanoma group, where nearly all of the SN+ patients are subsequently reclassified to the AJCC IIIA group and the prognosis remains excellent, regardless.^1^

### Risk of Sentinel Node Micrometastasis in the pT1b/pT2a Cohort

An initial analysis of our data demonstrated that lymphovascular invasion (LVI; syn. angiolymphatic invasion) had approximately 2.5 times the rate of SN+ compared with the rest of the cohort (*p* < 0.001), which is consistent with previous findings.^3,19–21^ This is a clear indication for SNB, yet the incidence of LVI was very low (2.0%), which makes this a poor stratifying variable for the early-stage melanoma cohort generally. Further analysis of the cohort revealed that SN+ was significantly associated with younger age, increasing Breslow thickness (or T-stage), increasing mitotic rate, primary location and tumor subtype. These results are consistent with the literature and are not necessarily specific to the early-stage group.^1,20–22^

Most current national guidelines use an internationally accepted threshold risk of 5% SN+ rate as an indication for performing SNB.^2,23^ In the current study, the overall SN+ rate was 7.6% in the pT1b cohort, 12.8% in the pT2a cohort, and 11.4% in the early-stage melanoma group as a whole. These data would seem to suggest that all patients with early-stage invasive melanoma should be offered SNB, yet the 5% threshold is arbitrary, derived from an era where systemic therapy was ineffective and the only successful treatment available at the time was surgery.^4^ In the modern paradigm, this threshold may need to be reconsidered, particularly when the outcome for ~90% of the cohort is a negative SNB. For two-thirds (278/404; 68.8%) of the SN+ cohort, the outcome is observation, according to current NCCN melanoma treatment guidelines.

Mitotic rate has been employed as a stratifying variable in the pT1b subgroup with successive iterations of the AJCC classification for melanoma,^1,9,11^ and by extension, as an indication for performing SNB. Other retrospective cohorts have investigated mitotic rate as a predictor of SNB status and outcome, and whilst there is no consistent value cutpoint, the previous literature consistently reports that the risks of both survival and SN status are directly proportional to mitotic rate.^1,22,24^ Data from this study demonstrated that a higher mitotic rate was significantly correlated with increasing Breslow thickness, and high mitotic rate tumors (MR >1/mm^2^) predominated in the T2 group. In our cohort, the median difference in Breslow thickness between the SN+ and SN− patients was statistically significant but not clinically relevant (1.2 vs. 1.3 mm; δ = 0.1 mm; *p* < 0.001). Accordingly, when considering the low-risk melanoma cohort (pT1b-pT2a) as a whole, it is reasonable to suggest that mitotic rate should be used as the leading primary tumor characteristic for predicting SN status.

### Nodal Disease Burden

Recently, several, prospective, randomized, controlled trials have shown clinical benefit of adjuvant systemic therapy, in terms of recurrence-free survival, for patients with metastatic melanoma.^7,25,26^ As a result, most modern clinical guidelines recommend it for high-risk resected metastatic melanoma.^2,27,28^ Whilst the main factor for offering adjuvant systemic therapy is based on the risk of recurrence and/or death from melanoma, the risk of toxicity from the systemic therapy may outweigh the potential benefits of the treatment, and the patient requires careful counseling accordingly. Our data confirm that the N-stage is not helpful as a determining factor for deciding whether to offer adjuvant systemic therapy in the early-stage melanoma patients with SN+ disease. The ongoing phase III adjuvant systemic therapy clinical trials for SN+ patients have, arbitrarily, only included those with a deposit greater than 1 mm, as these were judged to be a higher risk subgroup at trial design.

Data from this study has shown that the proportion of high-risk SNB metastases (>1 mm in maximum diameter) arising from low-risk primary melanomas was 31.3% (132/422). Multivariate analysis indicated that the only variable independently predictive of high-risk SN+ disease was mitotic rate. The odds of pT1b-pT2a primary melanomas with MR >1 mm^2^ being staged with a high-risk SN+ were almost twice those of melanomas with MR = 0-1/mm^2^. Furthermore, 77.8% (98/126) of all high-risk SN+ metastases arose from primaries with MR >1/mm^2^.

Likelihood ratios are useful to aid decision making when determining whether to undertake a test. Rather than predicting the chances of having a disease, using Bayes’ theorem, the ratio updates the clinician as to chances their patient has the disease in question by the magnitude of the likelihood ratio value. Assessment of the likelihood ratios in Table 2 for the mitotic rate categories indicates that, for the MR = 0/mm^2^ group, the ratio is 0.42 (95% CI: 0.29-0.60), indicating a small to moderate decrease in the likelihood of SN+. In addition, when assessing for likelihood of high-risk nodal disease, MR = 0/mm^2^ decreases that likelihood by a moderate to large amount (LR = 0.23; 95% CI: 0.1-0.5). Conversely, the likelihood SN+ and high-risk SN+ are both increased with a MR>1/mm^2^, although this change would be considered minimal from the underlying pretest probabilities of 11.4% and 3.6%, respectively. The likelihood ratio of the MR = 1/mm^2^ group does not change the odds of SN+ in a clinically relevant manner.

On one level, these data would suggest that clinicians targeting maximum potential from their SNB services would be well-served by prioritizing patients with MR >1/mm^2^ and for considering clinical surveillance of those patients with MR = 0/mm^2^. Unfortunately, the paradox remains that non-invasive techniques, especially ultrasound, are very poor at identifying nodal tumor deposits less than 1 mm in maximum diameter, even when performed contemporaneously with the SN-localization procedure immediately before SNB.^29^ Furthermore, data from the MSLT-1 study^4^ was highly suggestive of the phenomenon of disease progression within the metastatic sentinel nodes, and for a small subgroup of patients, excision of the metastatic focus in the sentinel node may be therapeutic in addition to diagnostic. The theoretical concern remains, therefore, that the significantly smaller proportion of patients with low-risk primary melanomas who are not offered SNB may be clinically disadvantaged with ultimately poorer outcomes in the longer term. With these data, clinicians deciding on prioritizing their services may wish to consider the risk of missing a SN metastasis in a melanoma with a very low likelihood of SN+, who may potentially still be salvaged by surgery and/or systemic therapy at a later date, against the value of SNB to the residual, much larger group who are SN−, who cannot benefit clinically from the procedure yet approximately 10% will develop a recurrence within 5 years. Clinical decision-making algorithms for calculating the threshold for offering a diagnostic test, considering the harms of under- and overtreating patients, and the benefits of correctly identifying treatable disease are potentially useful in this scenario,^30,31^ although ultimately setting the threshold for offering sentinel node biopsy will be the responsibility of national guidelines committees.

### Limitations

We acknowledge that the current study has several limitations, which arose from including multiple databases that were not uniformly aligned in data collection, and from the lack of a centralized pathological review of all cases, which was not practical. The classification of the host response to the primary tumor by the presence or absence of tumor infiltrating lymphocytes is well recognized as a predictor of SN status and patient outcome,^32^ but this data was not collected uniformly across the investigating centers. Similarly, extracapsular spread (syn. extracapsular extension) is recognized as a significant predictor of high-risk sentinel node metastases,^33^ but these data were not collected consistently across the units to allow analysis in the current dataset.

## Conclusions

In this current study, we have undertaken a large retrospective analysis of the outcome of sentinel node biopsy from 3,610 patients with early-stage primary cutaneous melanoma treated in seven, high-volume, cancer centers across three continents. The results would suggest that further research and a reappraisal of the role of SNB is required for a significant proportion of patients with early-stage invasive primary melanomas, because virtually all SN+ patients are restaged to AJCC IIIA. In the early-stage invasive melanoma group, a mitotic rate of >1/mm^2^ identifies patients with increased likelihood of SN metastasis and the greater proportion of SN+ who may be considered for adjuvant systemic therapy according to NCCN guidelines. The role of SNB for tumors with a mitotic rate of 1/mm^2^ tumors needs further clarification.
